# How significant is the ‘significant other’? Associations between significant others’ health behaviors and attitudes and young adults’ health outcomes

**DOI:** 10.1186/1479-5868-9-35

**Published:** 2012-04-02

**Authors:** Jerica M Berge, Rich MacLehose, Marla E Eisenberg, Melissa N Laska, Dianne Neumark-Sztainer

**Affiliations:** 1 Department of Family Medicine and Community Health, University of Minnesota Medical School, Minneapolis, USA; 2 Division of Biostatistics, University of Minnesota, Minneapolis, USA; 3 Division of Epidemiology and Community Health, University of Minnesota, Minneapolis, USA; 4 Division of Adolescent Health and Medicine, Department of Pediatrics, University of Minnesota, Minneapolis, USA; 5 Department of Family Medicine and Community Health, Phillips Wangensteen Building, 516 Delaware Street SE, Minneapolis, MN, 55455, USA

**Keywords:** Romantic relationships, Obesity, Dietary intake, Physical activity, Young adults

## Abstract

****Background**:**

Having a significant other has been shown to be protective against physical and psychological health conditions for adults. Less is known about the period of emerging young adulthood and associations between significant others’ weight and weight-related health behaviors (e.g. healthy dietary intake, the frequency of physical activity, weight status). This study examined the association between significant others’ health attitudes and behaviors regarding eating and physical activity and young adults’ weight status, dietary intake, and physical activity.

****Methods**:**

This study uses data from Project EAT-III, a population-based cohort study with emerging young adults from diverse ethnic and socioeconomic backgrounds (n = 1212). Logistic regression models examining cross-sectional associations, adjusted for sociodemographics and health behaviors five years earlier, were used to estimate predicted probabilities and calculate prevalence differences.

****Results**:**

Young adult women whose significant others had health promoting attitudes/behaviors were significantly less likely to be overweight/obese and were more likely to eat ≥ 5 fruits/vegetables per day and engage in ≥ 3.5 hours/week of physical activity, compared to women whose significant others did not have health promoting behaviors/attitudes. Young adult men whose significant other had health promoting behaviors/attitudes were more likely to engage in ≥ 3.5 hours/week of physical activity compared to men whose significant others did not have health promoting behaviors/attitudes.

****Conclusions**:**

Findings suggest the protective nature of the significant other with regard to weight-related health behaviors of young adults, particularly for young adult women. Obesity prevention efforts should consider the importance of including the significant other in intervention efforts with young adult women and potentially men.

## Background

Previous research has suggested that being involved in a committed relationship, or having a ‘significant other,’ improves individuals’ physical and psychological health and decreases mortality [1-5]. For example, longitudinal and cross-sectional studies examining individuals’ management of diabetes indicate that patients who included significant others in their care had significantly better hemoglobin A1C levels compared to those who did not [6,7]. Similarly, research on chemical dependency, depression, and compulsive gambling has suggested that involving a significant other in treatment increases the likelihood of treatment success [8]. Less is known about how health behaviors and attitudes of a significant other impact weight-related health behaviors, such as physical activity and daily dietary intake patterns, which can contribute to weight change over time.

According to family systems theory, the interactions that occur within romantic relationships are reciprocal [9,10]. That is, each partner is shaping and being shaped by the other partners’ actions (e.g. via support, modeling). These mutual influencing patterns may give particular insight into the behaviors that ultimately determine dietary intake and physical activity in young adults. For example, healthful dietary intake modeled by a significant other may potentially influence a young adult partner to engage in healthful eating as well.

Prior research has not determined whether the health behavior benefits of having a significant other apply to young adult partners. One study showed that young adults in committed romantic relationships had fewer mental health problems and were less likely to be overweight/obese [11], whereas another study showed entry into romantic partnership was associated with obesity [12]. These conflicting results may be due to differences in study design and sample characteristics. For example, one study was cross-sectional with a small sample size and lack of diversity [11], while the other was a large, longitudinal study [12]. Thus, to clarify inconsistencies in previous research findings, further research is needed to understand the role that a significant other’s health attitudes and behaviors play in young adults’ weight and weight-related health behaviors using a large racially/ethnically and socio-economically diverse sample. Furthermore, previous research on chronic health conditions has suggested that men benefit more from having a significant other than women [4,5,13-15]. Thus, it is also important to look at how men and women experience the significant other’s influence on weight and weight-related health behaviors, particularly among emerging young adults.

Given the increase in prevalence of obesity among young adults in the US [16], investigating whether significant others’ health behaviors and attitudes are associated with similar behaviors in young adults is important in identifying protective factors for adult obesity and in pinpointing targets for obesity prevention. The current study addresses these issues by answering the following research question: Is there an association between significant others’ health behaviors and attitudes and young adults’ dietary intake, frequency of physical activity, and weight status? We hypothesized that young adults who have significant others who care about, and engage in, healthy eating and activity will eat more fruits and vegetables, engage in more physical activity and be non-overweight/obese.

## **Methods**

### **Study design and sample**

Data for this analysis were drawn from Project EAT-III (Eating and Activity in Teens and Young Adults), the third wave of a population-based study designed to examine weight and weight-related outcomes among young adults. At baseline (Time 1, 1998–1999), a total of 4,746 middle school and high school students at 31 public schools in the Minneapolis/St. Paul metropolitan area of Minnesota completed surveys and anthropometric measures [17,18]. Five years later (Time 2, 2003–2004), for Project EAT-II, original participants were mailed follow-up surveys to examine changes in their eating patterns, weight control behaviors, and weight status as they progressed through adolescence [19,20]. Project EAT-III was designed to follow-up on participants again in 2008–2009 as they progressed from adolescence to young adulthood.

A total of 1,030 men and 1,257 women completed the Project EAT-III survey, representing 66.4% of participants who could be contacted (48.2% of the original school-based sample). Their mean age was 25.4 (sd = 1.5) The survey was pre-tested in focus groups and one-week test-retest reliabilities of the survey items were examined in a sample of 66 young adults. Additional details of the study and survey design have been reported elsewhere [21]. All study protocols were approved by the University of Minnesota’s Institutional Review Board.

Because attrition from the baseline sample did not occur at random, in all analyses, the data were weighted using the response propensity method [22]. Response propensities were estimated using a logistic regression of response at Time 3 on a large number of predictor variables from Project EAT-I. The weighting method resulted in estimates representative of the demographic make-up of the original school-based sample, thereby allowing results to be more fully generalizable to the population of young people in the Minneapolis/St. Paul metropolitan area. The current analysis included 1212 young adults who indicated they had a significant other, were neither pregnant nor breastfeeding, and who had reported health behavior outcome measures (BMI, fruits and vegetable intake, frequency of physical activity) at Time 2 ( 1).

**Table 1:** Characteristics and behaviors of EAT III young adults who have significant others at Time 3, (n = 1212)

	**Male Young Adult n = 519**	**Female Young Adult n = 693**
Overweight/Obese* (BMI ≥ 25)	55.7%	41.1%
**Daily intake of FV ≥ 5 servings/day**	23.4%	29.9%
Active > 3.5 hrs/wk*	54.6%	41.3%
**Age**	25.4 (1.6)	25.3 (1.7)
**Race**		
**White**	50.8%	47.5%
**Black**	15.5%	14.3%
**Hispanic**	5.6%	5.6%
**Asian**	21.1%	21.4%
**Mixed/Other**	7.0%	11.3%
**Educational Attainment**		
**Less than high school**	3.3%	4.0%
**High school or GED**	41.4%	32.1%
**Vocational School**	13.0%	15.4%
**Associate Degree**	12.1%	14.0%
**Bachelor Degree**	27.3%	30.2%
**Graduate Degree**	3.0%	4.3%

* Males and females were significantly different on these variables with p < .05.

### **Measures**

#### ***Dependent variables***

##### *Significant other status*

Significant other status was self-reported by young adult participants at Time 3. Young adults were asked the following question adapted from a previous measure [23] (yes/no): “Do you have a significant other (for example, boyfriend/girlfriend, spouse, partner)?”

##### *Significant others’ health behaviors*

Significant others’ health behaviors were assessed by two self-report items adapted from a previous measure [24] at Time 3: (1) “My significant other often plays sports or does something active,” and (2) “My significant other and I like to do active things together.” Both questions had response options ranging from “strongly disagree” to “strongly agree” on a 4-point scale. These items were dichotomized into strongly disagree/disagree (non-health promoting behaviors) and agree/strongly agree (health promoting behaviors). Test-retest values were high (Spearman r = 0.90 and 0.75 respectively).

##### *Significant other’s health attitudes*

At Time 3, young adults reported on their **s**ignificant others’ health attitudes with the following two items, adapted from a previous measure [24], “My significant other cares about eating healthy food” and “My significant other thinks it is important to be physically active”. The question on healthy eating had response options ranging from “not at all” to “very much” on a 4-point scale and was dichotomized into not at all/a little (non-health promoting attitudes) and somewhat/very much (health promoting attitudes). The question on physical activity had responses ranging from “strongly disagree” to “strongly agree” and was dichotomized into strongly disagree/disagree (non-health promoting attitude) and agree/strongly agree (health promoting attitude). Test-retest values were Spearman r = .77 and Spearman r = .69 respectively.

#### ***Independent variables***

Most of the dependent variables are measured at Time 2 and Time 3, in order to account for the effect of young adults’ previous health behavior five years earlier (Time 2). Adjusting for Time 2 behaviors reduces the likelihood that findings could be explained by previously established patterns of health behaviors and/or selection of a partner with similar habits.

##### *Body mass index (BMI)*

Height and weight among young adults were assessed by self-report at Time 2 and 3, which has been shown to be highly correlated with objectively measured values in adults [25-28]. BMI was calculated using the standard formula, weight (kg)/height (meters)^2^. In a validation study among a sub-sample of 127 Project EAT-III participants, the correlation between measured and self-reported BMI values was high (r = 0.95). Centers for Disease Control and Prevention cut-points were used to categorize participants into those who were normal weight (BMI < 25) or overweight/obese (BMI ≥ 25).

##### *Fruit and vegetable intake*

Young adults’ fruit and vegetable intake was assessed at Time 2 and Time 3 using a food frequency questionnaire. At Time 2, the 149-item Youth and Adolescent Food Frequency Questionnaire (YAQ) was used [29-31]. At Time 3, when adolescents had transitioned into young adulthood, a semi-quantitative food frequency questionnaire (FFQ 2007 grid form) was used [32]. For the current analysis, Time 3 self-reported servings of fruits (excluding fruit juice) and vegetables (excluding french fries) were dichotomized at ≥5 servings per day, based on dietary guidelines [33]. Previous studies have reported reliability and validity of intake estimates [34-36].

##### *Physical activity*

Physical activity questions were adapted from the Godin Leisure-Time Exercise Questionnaire [32]. Young adults were asked at Time 2 and Time 3, “In a usual week, how many hours do you spend doing the following activities:” (1) strenuous exercise (e.g. biking fast, aerobic dancing, running, swimming laps) (2) moderate exercise (e.g. walking quickly, gymnastics, baseball, skateboarding, easy bicycling). Response options ranged from “none” to “6+ hours a week”. Young adults’ self-reported hours of moderate to vigorous physical activity was dichotomized at ≥ 3.5 hours per week, based on current recommendations of 30 minutes of physical activity on most days [37].

#### ***Control variables***

Gender, age, race/ethnicity and educational attainment were assessed by self-report at Time 3. Race/ethnicity was assessed with one survey item: “Do you think of yourself as 1) white, 2) black or African-American, 3) Hispanic or Latino, 4) Asian-American, 5) Hawaiian or Pacific Islander, or 6) American Indian or Native American” and respondents were asked to check all that apply. Those reporting more than one response were coded as “mixed/other” youths. As few participants identified their background as “Hawaiian or Pacific Islander” or “Native American,” these youth were included in the category “mixed/other.” Highest level of educational attainment was assessed using the following question, “What is the highest level of education that you have completed?” Response options included: less than high school, high school/GED, vocational/technical school, associate degree, bachelor degree, graduate or professional degree [38].

#### ***Statistical analyses***

Chi-square and t-tests were used to compare dependent variables and demographic characteristics across gender. Separate multivariable logistic regression models were fit for each of the three dichotomous outcomes (overweight/obese, fruit and vegetable intake of 5 servings or more, and weekly physical activity of 3.5 hours or more) and each of the four dichotomous significant other health measures for a total of 12 regression models. All regression models were adjusted for participant age, educational attainment, and race/ethnicity, and were stratified by sex. Sex stratification was performed a priori due to previous research findings showing sex differences in BMI, fruit and vegetable intake, and physical activity in young adults **[**4,5,13-15]. In addition, stratifying by sex allowed for more flexible regression models since each adjustment variable included in the model could have a different point estimate for men and women. To account for the effect of previous health behavior, regression models were adjusted for each continuous health behavior outcome level at Time 2, thus reducing the likelihood that findings could be explained by previously established patterns of health behaviors and/or selection of a partner with similar habits.

Estimated coefficients from the regression models were used to calculate predicted prevalence estimates of the outcome for men and women at the mean level of continuous covariates in the model and the most common value of categorical covariates. Prevalence differences among men and women were computed as the difference between those whose significant other had a health promoting behavior/attitude and those whose significant other had a non-health promoting behavior/attitude. Differences in predicted prevalence estimates (i.e. prevalence differences or PD) were also calculated to estimate the effect of significant other’s attitudes on the “average” person in this cohort. Standard errors and 95% confidence intervals were calculated using the delta method [39]. All p-values have been reported in the text, in light of the multiple comparisons that could inflate the possibility of Type I error. All analyses were conducted using Stata (version 10.1, 2009, College Station, TX).

## **Results**

### **Descriptive statistics**

Young adult men were more likely to have a BMI ≥ 25 than women. Men were also more likely to engage in at least 3.5 hours of moderate or vigorous physical activity each week than women. Young adult men and women did not differ by fruit and vegetable intake or by sociodemographic characteristics (Table 1).

### **Associations between significant others’ health behaviors and attitudes and young adults’ health outcomes**

#### ***Young adult weight status***

After adjusting for adolescent weight status (i.e., Time 2) and sociodemographic characteristics, young adult women who reported that their significant other thought being physically active was important had a significantly lower prevalence of overweight/obesity compared to women who reported that their significant other thought physical activity was not important (47.1% versus 62.1%, respectively); this represented a difference between the two prevalence estimates of −15.1% (p = .045), as shown in Table 2. Similarly, the prevalence of overweight/obesity among women who reported that their significant other played sports or did something active was significantly lower than the prevalence of overweight/obesity among women who reported that their significant others were not active (PD:-14.1%; p = .023). There were no statistically significant associations between significant other health attitudes/behaviors and weight status for men.

**Table 2:** Significant others’ health behaviors and attitudes and young adults’ overweight/obese status* at Time 3

	**Male Young Adult**	**Female Young Adult**
	**Prevalence of Overweight/Obesity**	**Prevalence of Overweight/Obesity**
**Significant Others’ Behaviors & Attitudes**	**(n = 515)**	**(n = 688)**
**Cares About Healthy Eating:**		
**Not at all/Little**	60.9%	54.1%
**Somewhat/Very Much**	62.2%	47.4%
PD** (95% CI)	1.3% (−14.1%, 16.7%)	−6.8% (−19.9%, 6.4%)
**Thinks it is Important to be Physically Active:**		
**Strongly disagree/disagree**	50.6%	**62.1%**
**Agree/strongly agree**	64.8%	**47.1%**
PD** (95% CI)	14.3% (−0.7%, 29.3%)	−**15.1% (−29.8%, -0.3%)**
**Plays Sports or Does Something Active:**		
**Strongly disagree/disagree**	62.1%	**59.1%**
**Agree/strongly agree**	62.1%	**45.0%**
PD** (95% CI)	0.0% (−12.2%, 12.1%)	−**14.1% (−26.3%, -1.9%)**
**We do Active Things Together:**		
**Strongly disagree/disagree**	69.6%	55.5%
**Agree/strongly agree**	59.9%	48.4%
PD** (95% CI)	−9.6% (−21.8%, 2.5%)	−7.1% (−20.4%, 6.1%)

* All regression models adjusted for race, educational attainment, age and BMI at time 2.

**PD: Prevalence Difference, the difference between the predicted prevalences and their 95% confidence interval.

#### ***Young adult fruit and vegetable intake***

After adjusting for Time 2 adolescent fruit and vegetable intake and sociodemographic characteristics**,** young adult women had a significantly higher prevalence of eating five or more daily servings of fruits and vegetables if they reported having a significant other who cared about healthy eating (PD:11.5%; p = .009) or thought it was important to be physically active (PD:11.1%; p = 0.018), compared to women who reported that their significant other did not have a health promoting attitude (Table 3). There were no statistically significant associations between significant other health attitudes/behaviors and fruit and vegetable intake for men.

**Table 3:** Significant others’ health behaviors and attitudes and young adults’ fruit and vegetable intake (cut-point ≥ 5 servings/day)* at Time 3

	**Male Young Adult**	**Female Young Adult**
	**Prevalence eating ≥ 5 fruit/vege. per day**	**Prevalence eating ≥ 5 fruit/vege. per day**
**Significant Others’ Health Behaviors & Attitudes**	**(n = 458)**	**(n = 627)**
Cares About Healthy Eating:		
Not at all/Little	19.8%	**18.8%**
Somewhat/Very Much	23.9%	**30.3%**
PD** (95% CI)	4.1% (−7.7%, 15.8%)	**11.5% (2.8%, 20.1%)**
Thinks it is Important to be Physically Active:		
Strongly disagree/disagree	17.7%	**16.8%**
Agree/strongly agree	24.2%	**27.9%**
PD** (95% CI)	6.5% (−5.0%, 18.1%)	**11.1% (1.9%, 20.3%)**
Plays Sports or Does Something Active:		
Strongly disagree/disagree	19.3%	20.5%
Agree/strongly agree	25.7%	28.6%
PD** (95% CI)	6.5% (−3.0%, 16.2%)	8.1% (−0.6%, 16.8%)
We do Active Things Together:		
Strongly disagree/disagree	19.7%	21.8%
Agree/strongly agree	23.3%	27.0%
PD** (95% CI)	3.5% (−6.8%, 13.9%)	5.2% (−4.0%, 14.3%)

* All regression models adjusted for race, educational attainment, age and fruit and vegetable intake at time 2.

**PD: Prevalence Difference, the difference between the predicted prevalences and their 95% confidence intervals.

#### ***Young adult physical activity***

After adjusting for Time 2 adolescent physical activity and sociodemographic characteristics**,** young adult women had a significantly higher prevalence of engaging in at least 3.5 hours per week of physical activity if they reported that their significant others thought it was important to be physically active (PD:21.7%; p = 0.001), played sports or did something active (PD:14.4%; p = 0.003) or did active things together with them (PD:14.3%; p = 0.004), compared to women who reported that their significant others did not have health promoting behaviors and attitudes (Table 4). Similarly, young adult men had a significantly higher prevalence of engaging in at least 3.5 hours per week of physical activity if they reported having significant others who thought it was important to be physically active (PD:16.0%; p = 0.018), played sports or did something active (PD:13.5%; p = 0.013), or did active things together with them (PD:15.5%; p = 0.015), compared to men who reported that their significant other did not have health promoting behaviors and attitudes.

**Table 4:** Significant others’ health behaviors and attitudes and young adults’ physical activity (cut-point ≥ 3.5 hrs./week)* at Time 3

	**Male Young Adult**	**Female Young Adult**
	**Prevalence of physical activity ≥ 3.5 hrs./wk.**	**Prevalence of physical activity ≥ 3.5 hrs./wk.**
**Significant Others’ Health Behaviors & Attitudes**	**(n = 518)**	**(n = 693)**
Cares About Healthy Eating:		
Not at all/Little	57.6%	42.8%
Somewhat/Very Much	54.4%	49.8%
PD** (95% CI)	−3.2% (−16.9%, 10.4%)	7.0% (−2.1%, 16.2%)
Thinks it is Important to be Physically Active:		
Strongly disagree/disagree	**43.1%**	**29.6%**
Agree/strongly agree	**59.1%**	**51.2%**
PD** (95% CI)	**16.0% (2.7%, 29.3%)**	**21.7% (11.6%, 31.7%)**
Plays Sports or Does Something Active:		
Strongly disagree/disagree	**48.3%**	**37.9%**
Agree/strongly agree	**61.8%**	**52.3%**
PD** (95% CI)	**13.5% (2.8%, 24.2%)**	**14.4% (4.8%, 24.0%)**
We do Active Things Together:		
Strongly disagree/disagree	43.5%	**36.4%**
Agree/strongly agree	**59.0%**	**50.7%**
PD** (95% CI)	**15.5% (3.1%, 28.0%)**	**14.3% (4.5%, 24.1%)**

* All regression models adjusted for race, educational attainment, age and physical activity at time 2.

**PD: Prevalence Difference, the difference between the predicted prevalences and their 95% confidence intervals.

## **Discussion**

Results indicated that for young adult women, having a significant other who had health promoting attitudes and behaviors was associated with an increased likelihood of eating fruits/vegetables and engaging in physical activity, and a decreased likelihood of being overweight/obese, even after adjusting for young adult health behaviors five years earlier. For young adult men, having a significant other who had health promoting behaviors and attitudes was associated with an increased likelihood of engaging in physical activity, but was not associated with healthy eating and/or weight status. Taken together, these findings suggest that having a significant other with health promoting behaviors and attitudes may be protective against adult obesity and other weight-related health behaviors, particularly in young adult women.

Previous studies have shown that men generally benefit more from having a significant other than women [5,6,8,10]. In contrast, results from the current study suggest that young adult women may benefit more from significant other support for weight and weight-related health behaviors (e.g. physical activity and dietary intake habits), compared to young adult men. One explanation for this finding may be related to previous research in the fields of family science and psychology suggesting women are more focused on “relationship” or “connectedness” factors than men [40-42]. This emphasis on connectedness may have an important impact on health behaviors, in that women may pay attention to, or focus more on, the feelings or behaviors of their significant other more than men, thus, making women more likely to engage in similar behaviors, while men may engage in health behaviors regardless of their significant others’ modeling or health attitudes.

It may also be the case that women experience their significant other’s health promoting behaviors and attitudes as a type of “support system” in their own efforts to be healthy. This hypothesis is supported by weight-loss research that has shown that women identify the importance of having a significant other as an exercise partner when trying to increase daily exercise, keep weight off and make healthy dietary changes [43,44]. Thus, results of the current study are important to consider in regard to obesity treatment and prevention efforts with young adult women. Including a significant other in interventions may be important in creating supportive environments for health behaviors to occur. Also, assessing the level of health-related support from the significant other would be important in order to gauge the level of support that is available for the young adult women.

While having a significant other with health promoting behaviors/attitudes was not associated with decreased overweight/obesity or increased fruit and vegetable intake for young adult men, there was a significant association with increased physical activity. This is important to consider when targeting men in obesity prevention interventions. It may be the case that men are influenced by the significant other, but only through specific mechanisms such as physical activity.

This study has a number of strengths, including the use of a large, diverse, population-based, longitudinal cohort sample allowing for generalizability of study findings to other young adult populations from US metropolitan areas. However, findings from this study must also be interpreted in light of certain limitations. First, the survey utilized here did not assess for length of relationship. It is possible that the longevity of a relationship may be related to the strength of the influence of the significant other on young adults’ health behaviors. Further, only participant’s report of significant others’ health behaviors and attitudes were assessed. While this may be a limitation, it may also be equally important to assess. For example, research has shown that one’s perception of a romantic partner’s beliefs and behaviors versus measured beliefs and behaviors is more predictive of one’s own beliefs and behaviors [45]. Although adjustment for Time 2 health behaviors and BMI allowed us to reduce issues of unmeasured confounding due to the self-selection of a partner with similar health behaviors and weight status, these issues may not have been entirely eliminated and residual confounding may still exist. In addition, while adjusting for outcomes at Time 2 allows for a better understanding of the temporal relationship between significant others’ health attitudes and behaviors and young adults’ weight and weight-related behaviors by accounting for potential differences in the outcome measures previous to when the young adults had significant others (e.g. in adolescence/young adulthood), it does not entirely explain temporality due to the cross-sectional nature of this study.

Of note, the behaviors of the significant other have all been interpreted to be positive (i.e. support for healthy eating), but it may also be the case that some significant others are more controlling over their partners' weight and health behaviors, or that they communicate negative attitudes about weight gain. These types of significant other behaviors may also result in healthier diets, exercise, and weight status in the partner, but not through supportive means. Given findings from the current study, suggesting the importance of significant others, future research should explore the types of comments, their context and how they are perceived by the recipient.

## **Conclusions**

Results from this study suggest that young adults, particularly women, may benefit more from having a significant other in relation to health behaviors which contribute to weight and weight change over time (e.g. physical activity and dietary intake habits) compared to men**.** Further research should explore additional aspects of relationships with significant others such as commitment level (e.g. dating, cohabitating, married) and length of relationship to further understand the influence of the significant other on young adults’ weight and weight-related outcomes.

## **Competing interests**

All authors have no conflicts of interest, nor financial disclosures to report.

## **Authors' contributions**

All co-authors made a substantial contribution to the paper. JMB conceptualized the paper, interpreted the data analysis, and wrote all drafts of the paper. DNS is the principal investigator of Project EAT and assisted in conceptualizing the paper and critically revised the paper. RM conducted the data analysis and interpretation. ME assisted with the conceptualization of the paper, interpretation of the data, and critically revised the paper. ML assisted the interpretation of the data and critically revised the paper. All authors read and approved the final manuscript.
